# Higher COVID‐19 risk for patients with cancer

**DOI:** 10.1002/mco2.21

**Published:** 2020-10-12

**Authors:** Jincheng Yang, Zhe Chen, Bei Jia

**Affiliations:** ^1^ Department of Pharmacy, National Cancer Center/National Clinical Research Center for Cancer/Cancer Hospital Chinese Academy of Medical Sciences and Peking Union Medical College Beijing China

Dear Editor,

The whole world has been experiencing the outbreak of COVID‐ 19 (corona virus disease‐19) since the end of 2019. By July 1, 2020, the rapid spread virus had caused globally cumulative over 10 million confirmed cases (over 500 000 death cases) from WHO report. As for reported cancer patients in *The Lancet Oncology*, it hypothesized about risk factors in that a higher rate of smoking history as an independent risk factor in severe cases, inducing the uncertain correlation between cancer and COVID‐19.^1^

Human angiotensin‐converting enzyme 2 (hACE2), a monocarboxypeptidase and a pivotal component of the renin‐angiotensin system (RAS), is recognized as the entry receptor by COVID‐19.^2^ RAS is composed of two axes, in which ACE2 degrades angiotensin II to produce Ang(1‐7). ACE2 is expressed less than 1% in lung cells, and more than 80% in type II alveolar cells.^3^ Enhanced ACE2 expression is considered to be protective in patients with diabetes, cardiovascular disease, and cancer.

First, ACE2 inhibited the growth of lung cancer, metastasis of prostate cancer, and angiogenesis of breast cancer, while it resulted in better prognosis in hepatocellular carcinoma. Additionally, some chemotherapeutic agents (gemcitabine and cisplatin) significantly increased ACE2 levels in lung cancer treatment in vitro.^4^ A clinical trial indicated that lower serum ACE2 levels correlated with higher postoperative morbidities and in‐hospital mortality ratios after surgery of major pulmonary resection in non‐small cell lung cancer (NSCLC) patients.^5^ Therefore, increasing ACE2 expression might be one benefit method and/or outcome for cancer treatment. However, such approach to increase ACE2 also increases more entrance “gates” for COVID‐19.

Second, the effect of nicotine upon ACE2 is still under disputation at animal experimental level. Therefore, smoking might not be a certain risk factor for COVID‐19.

Additionally, authors who published researches on database analyses considered reports from public databases sometimes showed controversial consequences. Sample size and the purity of ACE2‐expressing cells in the selected samples would influence final conclusions, as ACE2‐positive cells actually hold limited part in lung tissues. The expression level and expression pattern of hACE2 in different tissues might be critical for the susceptibility, symptoms, and outcome of COVID‐19 infection. Thus, conclusions from only one analytical article are not as authentic enough as that from animal and mechanical experiments.

Overall, cancer patients are at higher risk of COVID‐19 than other individuals as the result of ACE2‐upgraded effect, besides the patient immunosuppressive state and other side effects from oncotherapy (Figure 1).

**FIGURE 1:**
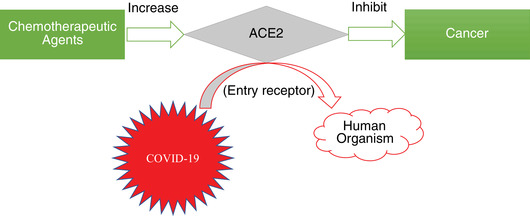
Effect of ACE2 between cancer patient and COVID‐19

Therefore, we propose major strategies for cancer patients besides common shelters for individuals. First, cancer patients should delay surgery at disease stable states and add interval chemotherapies. Second, most cancer patients should decrease chemotherapeutic dosage or prolong chemotherapeutic period without influencing tumor treatment and prognosis, or take long‐term oral therapeutic regimes. Third, local examination and online diagnosis should be promoted when new symptoms emerge; take whole protection during off‐line diagnosis. Fourth, take necessary nutrition care, rest, and physical exercises at home.

## CONFLICT OF INTEREST

The authors declare no potential conflict of interest.
